# 

**Published:** 1854-01-01

**Authors:** 


### THE JOURNAL

OF

### PSYCHOLOGICAL MEDICINE

AND

### MENTAL PATHOLOGY.

EDITED BY

FORBES WINSLOW, M.D. D.C.L.

LATE PRESIDENT OF THE MEDICAL SOCIETY OF LONDON.

VOL. VII.

[illegible] Office. [illegible]ARY. 22057 Washington, D.C.

LONDON:
JOHN CHURCHILL, NEW BURLINGTON STREET.
MDCCCLIV.

W
JO 851 N

LONDON:
SAVILL AND EDWARDS, PRINTERS, CHANDOS STREET,
COVENT GARDEN.

### LETTSOMIAN LECTURES.

---

No. I.

### THE PSYCHOLOGICAL VOCATION OF THE PHYSICIAN.

*Delivered before the Medical Society of London, April 7, 1852.*

BY

FORBES WINSLOW, M.D. D.C.L.

### APPOINTMENTS.

William P. Kirkman, Esq., of the Suffolk County Lunatic Asylum, has been appointed Assistant Medical Officer to the Devon County Asylum, in the room of Dr. Manly, who resigned at Christmas.

Theodore S. G. Boisragon, M. D., Physician to the Cornwall County Lunatic Asylum.

